# Exploration of lifestyle interventions for individuals with knee osteoarthritis: A scoping review

**DOI:** 10.4102/sajp.v82i1.2370

**Published:** 2026-06-09

**Authors:** Bashir Bello, Fatima Y. Aliyu, Bashir Kaka, Ushotanefe Useh

**Affiliations:** 1 Department of Physiotherapy, Faculty of Allied Health Sciences, Bayero University, Kano, Nigeria; 2 Lifestyle Disease Research Entity, Faculty of Health Sciences, North-West University, Mafikeng, South Africa

**Keywords:** knee osteoarthritis, lifestyle intervention, exercise therapy, nutrition, scoping review, outcome measures, multimodal intervention

## Abstract

**Background:**

Knee osteoarthritis (OA) is a primary global cause of disability, with lifestyle interventions such as exercise and weight management serving as key conservative strategies. However, the diversity of these interventions and their assessment measures lack synthesis.

**Objectives:**

Our scoping review explored the range, characteristics and outcomes of lifestyle interventions for knee OA and identified the outcome measures used for assessment.

**Method:**

Adhering to Preferred Reporting Items for Systematic Reviews and Meta-Analyses Extension for Scoping Reviews guidelines and the Arksey and O’Malley framework, data were extracted from 36 studies, including 29 randomised controlled trials, two cohorts, three quasi-experimental studies, one qualitative study and one feasibility trial.

**Results:**

Across 4025 participants, interventions included physiotherapist-guided exercise, multicomponent programmes, digital eHealth platforms, aquatic training and dietary changes. While findings consistently showed improved physical function, reduced pain and enhanced quality of life, no single outcome measure was universally applied across all identified studies. Key barriers included low motivation, technical literacy and resource intensity.

**Conclusion:**

Lifestyle interventions are diverse and effective for improving clinical outcomes in knee OA. The absence of a standardised outcome measure highlights significant heterogeneity in how intervention efficacy is captured in research.

**Clinical Implications:**

These findings highlight the need for standardised, clinically meaningful outcome measures to improve comparability across studies and strengthen evidence synthesis. Greater consistency in how lifestyle interventions are evaluated will support translation into clinical practice and inform the design of future multicomponent programmes for individuals with knee OA.

## Introduction

Osteoarthritis (OA) is the most common form of arthritis worldwide, characterised by the progressive degeneration of joint cartilage and underlying bone, leading to pain, stiffness and functional limitations (Hunter & Bierma-Zeinstra 2019). The prevalence of OA has been increasing in tandem with global trends in ageing populations and rising obesity rates (Safiri et al. 2020). Estimates suggest that approximately 528 million people were living with OA in 2019, an increase of 113% since 1990, making it a leading cause of disability globally (Vos et al. 2020). The burden of OA is not confined to individual suffering but extends to high societal costs, including increased healthcare utilisation and productivity losses (Cross et al. 2014). Historically, the management of OA has focused heavily on pharmacological interventions, such as non-steroidal anti-inflammatory drugs and intra-articular corticosteroid injections, as well as surgical options like joint replacement in advanced cases (Bannuru et al. 2019). While these treatments can offer symptom relief, they do not address the underlying risk factors driving disease progression, nor do they come without side effects and complications (Fernandes et al. 2013). Consequently, there has been a growing recognition of the importance of non-pharmacological approaches, particularly lifestyle interventions, in managing OA holistically and sustainably.

Lifestyle interventions encompass a range of strategies aimed at modifying daily habits and behaviours to improve health outcomes. For individuals with OA, these interventions may include physical activity programmes, weight management, dietary modifications, self-management education and psychological support (Hawker 2019).

Clinical guidelines from major organisations, including the Osteoarthritis Research Society International and the American College of Rheumatology, strongly recommend that core treatments for OA should prioritise patient education and structured exercise alongside weight loss when appropriate (Bannuru et al. 2019). Physical activity, particularly aerobic and strengthening exercises, is consistently identified as a cornerstone of OA management (Fransen et al. 2015). Evidence indicates that exercise reduces pain, improves function and may even delay structural progression (Uthman et al. 2013). Yet, despite its well-established benefits, adherence to exercise regimens among individuals with OA remains suboptimal due to factors such as pain, fear of exacerbating symptoms, a lack of motivation and limited access to tailored programmes (Giardulli et al. 2025).

Weight management is another critical component, especially for individuals with knee and hip OA. Excess body weight increases joint loading, exacerbates pain and accelerates cartilage degeneration (Bliddal, Leeds & Christensen 2014).

Studies have shown that a modest weight reduction of 5% – 10% can significantly decrease pain and improve physical function in overweight or obese individuals with OA (Messier et al. 2013). However, sustainable weight loss requires long-term lifestyle changes, combining dietary modifications with physical activity and behavioural strategies (Christensen et al. 2007). Dietary interventions themselves are gaining attention, not only for their role in weight management but also for their potential anti-inflammatory effects (Méndez & Medina 2021). Diets rich in omega-3 fatty acids, antioxidants and polyphenols have shown promise in reducing OA-related inflammation and pain (Sanghi et al. 2009). However, the evidence base remains fragmented, and more rigorous studies are needed to establish specific dietary recommendations for OA patients. Beyond physical and nutritional aspects, psychological and social factors play a significant role in the lived experience of OA. Chronic pain and disability can lead to depression, anxiety and social isolation, which in turn may hinder engagement in beneficial lifestyle behaviours (Stubbs et al. 2016). Therefore, cognitive behavioural therapy, pain coping skills training and peer support groups are increasingly integrated into comprehensive OA management to address psychosocial barriers and promote self-efficacy (Somers et al. 2012).

Recent developments in knee OA management have emphasised the importance of delivering lifestyle interventions in real-world, patient-centred contexts. Approaches such as shared decision-making and tailoring interventions to individual values, preferences and readiness for change have led to increasingly diverse and adaptive programme designs (Hunter & Bierma-Zeinstra 2019). In parallel, the growing use of digital health strategies, including telerehabilitation and mobile health applications, has further expanded the modes of delivery and accessibility of these interventions (Hinman et al. 2020). While these advances are promising, they contribute to considerable variation in how lifestyle interventions are conceptualised, implemented and evaluated. This heterogeneity underscores the need for a comprehensive synthesis of the range, characteristics and outcome measures of lifestyle interventions for individuals with knee OA, which our scoping review aims to address.

Despite the abundance of individual studies and systematic reviews examining various lifestyle strategies for OA, a comprehensive mapping of the evidence remains necessary to identify gaps and guide future research, policy and practice for knee OA. Scoping reviews offer an effective methodological approach to collate and synthesise heterogeneous evidence, especially when the topic is complex and has not been comprehensively reviewed before (Peters et al. 2020). By systematically exploring the range and nature of lifestyle interventions for individuals with OA, our review aimed to examine the characteristics and components of lifestyle interventions for knee OA and to identify the specific outcome measures used to evaluate their efficacy.

## Research methods and design

### Study design

Our study employed a scoping review methodology guided by the framework proposed by Arksey and O’Malley (2005) and enhanced by the Joanna Briggs Institute (Peters et al. 2020). A scoping review was chosen to comprehensively map the breadth and depth of evidence on lifestyle interventions for individuals with OA, identify research gaps and inform future studies and clinical practice.

### Research questions

Our review was guided by the following research questions: (1) *What types of lifestyle interventions have been used for individuals with OA?*; (2) *What are the characteristics, components and delivery modes of these interventions?*; (3) *Which outcome measures are most frequently used to evaluate the intervention efficacy?* and (4) *What gaps exist in the current evidence base?*

### Eligibility criteria

The inclusion criteria for selecting studies were defined using the population, concept and context framework:

- **Population:** Adults (≥ 18 years) diagnosed with knee OA.
- **Concept:** Lifestyle interventions, including but not limited to exercise programmes, physical activity, weight management, dietary modifications, psychological interventions, self-management education and digital health solutions related to lifestyle behaviour change.
- **Context:** All healthcare and community settings. There were no restrictions on geographical location or the healthcare system.

**Types of sources:** Both quantitative (e.g. randomised controlled trials [RCTs], cohort studies, cross-sectional studies) and qualitative studies were eligible. Systematic reviews and meta-analyses were screened for relevant primary studies. Only articles published in English were included. Conference abstracts, commentaries and editorial pieces were excluded unless they presented original empirical data.

### Information sources and search strategy

A comprehensive search strategy was developed in collaboration with an experienced health sciences librarian.

The following electronic databases were searched from inception to June 2025: Medical Literature Analysis and Retrieval System Online (MEDLINE) (via PubMed), Google Scholar, Cumulative Index to Nursing and Allied Health Literature (CINAHL), Scopus and Cochrane Library.

The search combined keywords and Medical Subject headings (MeSH) terms related to OA (e.g. ‘osteoarthritis’, ‘OA’), lifestyle interventions (e.g. ‘exercise’, ‘physical activity’, ‘weight loss’, ‘diet’, ‘self-management’) and relevant synonyms. Boolean operators (‘AND’, ‘OR’) were used to maximise sensitivity. The full search strategies for the databases are provided in Online Appendix 1. In addition, the reference lists of included articles and relevant systematic reviews were hand-searched to identify additional studies not captured in the database search.

### Study selection

All identified records were imported into EndNote for reference management and then uploaded to Covidence for screening. Duplicates were removed automatically and verified manually.

**FIGURE 1:**
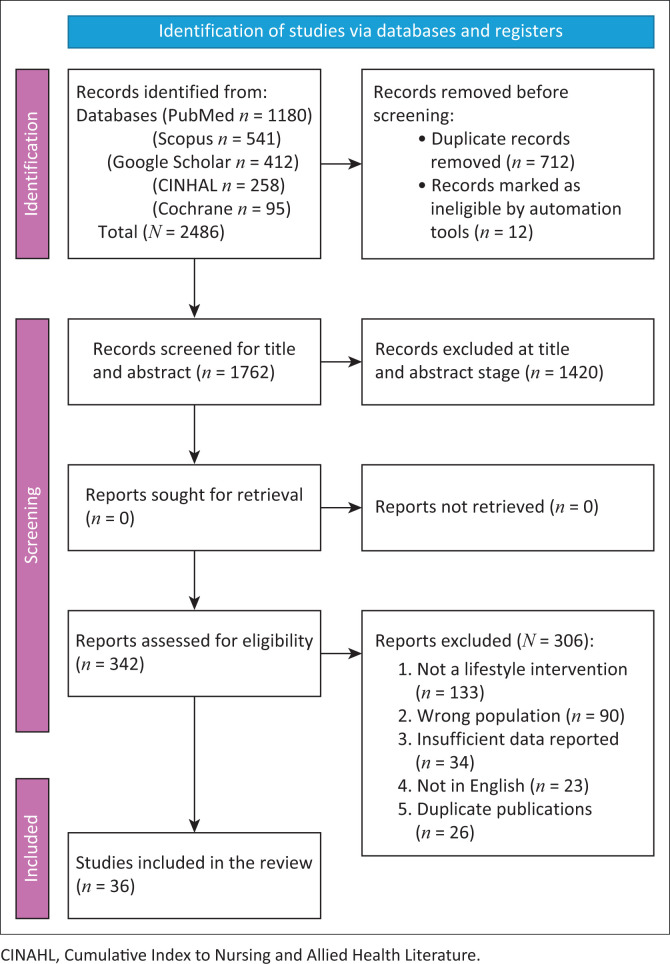
Flowchart of the scoping review screening process.

Title and abstract screening were conducted independently by two reviewers. Studies deemed potentially relevant underwent full-text screening, which was conducted independently by two reviewers. All screening decisions were made in agreement, without the need for adjudication by a third reviewer. The selection process is reported using the Preferred Reporting Items for Systematic Reviews and Meta-Analyses Extension for Scoping Reviews flow diagram (Tricco et al. 2016). See Figure 1 for full details.

### Data extraction

A standardised data extraction form was developed and pilot-tested on a sample of five studies to ensure consistency and comprehensiveness. Extracted data included: author(s), year, country, study design, sample size, participant demographics, interventions (type, duration, frequency, delivery mode, providers involved and setting), outcomes measured (pain, physical function, quality of life, adherence, patient satisfaction and other relevant outcomes), key findings (summary of results related to lifestyle intervention effectiveness or implementation) and conclusions as reported by the authors. Data extraction was conducted by two reviewers (authors Bashir Bello and Fatima Y. Aliyu) and checked for accuracy by a third reviewer (author, Bashir Kaka). Discrepancies were resolved through discussion.

### Data analysis and synthesis

The extracted data were collated and synthesised descriptively using tables and narrative summaries to map the range and characteristics of lifestyle interventions for OA. Interventions were categorised by type (e.g. exercise, diet, self-management) and delivery mode (e.g. in-person, digital, group-based). Outcomes were grouped thematically, and variations in intervention design and implementation were noted. The synthesis focused on highlighting commonalities, unique approaches and gaps in the evidence, without performing a formal assessment of methodological quality, as is consistent with scoping review methodology (Peters et al. 2020). No patients or members of the public were involved in the design, conduct or reporting of our scoping review.

### Ethical considerations

Ethical clearance to conduct our study was obtained from the Bayero University Kano Health Research Ethics Committee (No. NHREC/BUK-HREC/773/10/23II).

## Results

A total of 36 studies met the inclusion criteria for our scoping review, representing a diverse array of research designs including RCTs, quasi-experimental studies, cohort studies, feasibility trials, qualitative studies and narrative reviews. See Table 1 for a full description of the included studies. The included studies were published between 2009 and 2024 and conducted across various geographical regions, including Australia, the United States (US), the United Kingdom (UK), Saudi Arabia, Brazil, Pakistan and Nigeria. Most studies were RCTs with sample sizes ranging from 20 participants to 232 participants. The diversity of study populations and settings underscores a global effort to investigate lifestyle interventions for managing knee OA.

**TABLE 1:** Lifestyle intervention studies for managing knee osteoarthritis.

Author (year)	Study design	Country	Sample size	Intervention type	Outcome measures	Key findings	Barriers	Facilitators
Alghadir et al. (2019)	RCT	Saudi Arabia	60	Physiotherapy + education	WOMAC, ROM	Improved function and range of motion	Low motivation	Structured physio sessions
Alfieri et al. (2020)	RCT	UAE	90	Multicomponent lifestyle programme	Pain score, BMI	Improved pain and BMI	Compliance	Holistic approach
Bendrik et al. (2021)	Qualitative	US	20	Home exercise + coaching	Self-efficacy, pain score	Enhanced autonomy and lower pain	Isolation	Coach feedback
Bennell et al. (2017a)	RCT	Australia	148	Physiotherapist-guided exercise	WOMAC, SF-36	Improved pain and function	Resource intensity	Video delivery
Bennell et al. (2022)	RCT	Australia	193	Web-based pain coping skills + physiotherapy	WOMAC, PCS, SF-36	Online support boosted outcomes	Tech usability	Self-paced learning
Bennell et al. (2017b)	RCT	Australia	193	Web-based skills training	WOMAC, PCS	Improved coping and outcomes	Tech literacy	Accessible modules
Chen et al. (2021)	RCT	Taiwan	88	Balance training + Tai Chi	BBS, WOMAC	Improved balance and reduced OA symptoms	Session frequency	Cultural relevance
De Rooij et al. (2017)	RCT	The Netherlands	200	Tailored exercise therapy	EQ-5D, WOMAC	Better quality of life and pain reduction	Diversity in needs	Custom plans
Focht et al. (2022)	Cohort	US	102	Online education platform	Adherence, knowledge	Boosted health literacy	Digital fatigue	Interactive content
Gohir et al. (2021)	RCT	UK	140	Mediterranean diet + physio	Pain, QoL	Combined intervention yielded benefit	Diet adherence	Structured guidance
Gudbergsen et al. (2021)	RCT	Denmark	180	eHealth-supported OA programme	WOMAC, physical activity	Digital tool improved self-management and mobility	Digital literacy	User-friendly app design
Godziuk et al. (2023)	RCT	Poland	110	Exercise therapy with counselling	WOMAC, TUG	Sustainable improvements in mobility	Counselling consistency	Combined services
Harris et al. (2023)	RCT	US	70	Mobile app-based coaching	Pain VAS, steps	High engagement and physical activity	App literacy	Push notifications
Henriksen et al. (2023)	RCT	Norway	210	Group therapy + coping training	Coping scales, WOMAC	Stronger coping, better function	Group dropout	Social support
Hinman et al. (2020)	RCT	Australia	134	Telehealth physiotherapy	WOMAC, function scores	Positive clinical effects with remote delivery	Digital access	Accessible care
Hinman et al. (2017)	RCT	Australia	134	Tele-physiotherapy	WOMAC, SF-12	Similar outcomes as in-person therapy	Internet access	Remote flexibility
Holm et al. (2023)	RCT	Sweden	150	Cognitive functional therapy	PCS, disability index	Improved psychological outcomes	Therapist burden	Custom approach
Jorge et al. (2015)	RCT	Brazil	24	Resistance training (land vs. aquatic)	VAS, WOMAC	Aquatic training more effective for pain	Intensity limitations	Safe environment, guidance
Khachian et al. (2020)	Quasi-Exp.	Iran	85	Aquatic training	Pain, function	Improved mobility	Facility access	Fun format
Kaufman et al. (2022)	Cohort	US	75	Peer-led lifestyle Ed.	BMI, QoL	Small improvements in diet and pain	Low attendance	Peer motivation
Lange et al. (2009)	RCT	US	130	Tai Chi vs. stretching	Pain score, TUG	Tai Chi group improved significantly	Cultural unfamiliarity	Group dynamics
Lawford et al. (2023)	RCT	Australia	212	Remote strength training	WOMAC, strength test	Significant strength gains	Tech issues	Telecoaching
Messier et al. (2013)	RCT	US	150	Weight loss + exercise + diet	WOMAC, SF-36	Improved outcomes from combined approach	Lifestyle modification difficulty	Integrated plan
Mohammed and Rasool (2023)	RCT	Iraq	94	Mindfulness yoga	QoL, pain score	Reduced anxiety and pain	Scepticism	Mind-body alignment
Ojoawo et al. (2024)	RCT	Nigeria	60	Physiotherapy + education	WOMAC, SF-36, TUG	Significant improvement in physical function	Accessibility of follow-up	Culturally appropriate intervention
Rafiq et al. (2021)	RCT	Pakistan	100	Home-based rehab + diet education	VAS, WOMAC	Improved pain and function	Patient compliance	Home delivery, low cost
Sadeghi et al. (2022)	RCT	Iran	70	Nutritional + exercise counselling	WOMAC, QoL index	QoL and symptom relief enhanced	Limited counselling sessions	Holistic integration
Saleem et al. (2022)	RCT	Pakistan	120	Physical therapy with CBT	WOMAC, SF-36	CBT addition boosted mental and physical scores	Mental health stigma	Combined treatment acceptance
Saw et al. (2016)	RCT	Malaysia	88	Group exercise + peer support	WOMAC, SF-36, PAQ	Higher adherence and outcome sustainability	Group scheduling conflicts	Peer motivation
Sengul et al. (2022)	RCT	Turkey	76	Online exercise and advice	WOMAC, PSQI, EQ-5D	Improved pain, function, sleep	Tech issues	Remote access
Smith et al. (2023)	Quasi-Exp.	US	166	6-week exercise + education (OA-Fit)	KOOS, patient activation, PROMIS fatigue	Improvements in pain, function, fatigue, and patient activation sustained	Engagement over time	Scalable delivery, coach support
Somers et al. (2012)	RCT	US	232	CBT + lifestyle education	WOMAC, pain, depression, weight	Improved function, mood, and weight management	Psychosocial stress	Group support, psychological tools
Strath et al. (2020)	Feasibility trial	US	20	Behavioural exercise + OA education	Accelerometer PA, WOMAC, PROMIS	Improved PA and symptoms	Limited sample	Wearables, behaviour support
Taglietti et al. (2018)	RCT	Brazil	64	Aquatic exercise	WOMAC, SF-36, sit-to-stand	Improved pain, function, QoL	Pool access	Warm-water, low joint load
Walrabenstein et al. (2022)	Quasi-Exp.	The Netherlands	200	Lifestyle coaching + digital support	PA, diet, pain self-management	Behavioural change, empowerment	Tech-literacy gaps	e-Health, coaching
Wang et al. (2021)	RCT	China	120	Mindfulness-based Tai Chi vs. education	WOMAC, pain VAS, SF-12	Greater physical and mental function improvements	Adherence	Cultural fit, instructors

Note: Please see the full reference list of the article, Bello, B., Aliyu, F.Y., Kaka, B. & Useh, U., 2026, ‘Exploration of lifestyle interventions for individuals with knee osteoarthritis: A scoping review’, *South African Journal of Physiotherapy* 82(1), a2370. https://doi.org/10.4102/sajp.v82i1.2370

BBS, Berg Balance Scale; BMI, body mass index; CBT, cognitive behavioural therapy; EQ-5D, EuroQol 5-Dimension; KOOS, Knee Injury and Osteoarthritis Outcome Score; OA, osteoarthritis; PA, physical activity; PAQ, physical activity quotient; PCS, Pain Catastrophising Scale; PROMIS, patient-reported outcomes measurement information system; PSQI, Pittsburgh Sleep Quality Index; QoL, quality of life; RCT, randomised controlled trail; SF-12, 12-item short form survey; TUG, Timed Up and Go; VAS, Visual Analogue Scale; WOMAC, Western Ontario and McMaster Universities Osteoarthritis Index; ROM, range of motion; UAE, United Arab Emirates; US, United States; UK, United Kingdom; vs., versus; Quasi-Exp., quasi-experimental.

The studies examined a wide range of lifestyle interventions, including exercise therapy, dietary modification, psychological strategies, education and combinations of these modalities. Exercise-based interventions were the most prevalent, including resistance training (Taglietti et al. 2018), aquatic exercise (Carmona-Teres et al. 2015), Tai Chi (Sadeghi et al. 2023) and physiotherapist-led home exercise programmes (Hinman et al. 2020). Dietary interventions included structured weight loss programmes, Mediterranean diets and anti-inflammatory nutrition plans aimed at reducing OA-related symptoms (Messier et al. 2013; Rafiq, Abdul Hamid & Hafiz 2021). Psychological strategies were integrated in several studies, including cognitive behavioural therapy (Somers et al. 2012) and mindfulness-based approaches (Harris et al. 2023). Educational components were either standalone or embedded within multicomponent programmes, often delivered through in-person sessions or digital platforms (Lawford et al. 2023; Smith et al. 2023).

Intervention delivery methods varied considerably. Some programmes were conducted face-to-face in clinical or community settings, while others employed telehealth, mobile applications or online portals. For instance, Lawford et al. (2023) examined a telerehabilitation programme integrating physiotherapy with online education, while Holm et al. (2023) utilised a remotely delivered neuromuscular exercise intervention. Home-based and self-managed models were also reported, such as the digital exercise and education programmes evaluated by Bennell et al. (2017a) and De Rooij et al. (2017). Multidisciplinary teams, including physiotherapists, dietitians and psychologists, were commonly involved in delivering these interventions, particularly in trials combining physical and psychosocial support.

One of the objectives of our review was to identify the outcome measures used to assess these lifestyle interventions. Analysis of the data revealed that no single outcome measure was employed universally across all 36 identified studies. Instead, the authors utilised a broad array of validated tools to capture different dimensions of the disease. The Western Ontario and McMaster Universities Osteoarthritis Index (WOMAC) was the most frequently utilised tool for assessing pain and physical function. Other common clinical metrics included the Visual Analogue Scale for pain intensity, the Timed Up and Go (TUG) test for functional mobility and the Knee Injury and Osteoarthritis Outcome Score (KOOS). To measure quality of life and psychosocial impact, studies often employed the Short Form Health Survey (SF-36 or SF-12), the EuroQol-5D and the Pain Catastrophising Scale.

Across the included studies, the majority reported favourable outcomes associated with lifestyle interventions.

Exercise and education programmes consistently improved pain, function and quality of life among individuals with knee OA (Bennell et al. 2022; Taglietti et al. 2018). Multicomponent interventions that combined physical activity with education, psychological support or nutritional counselling yielded greater improvements in outcomes compared to single-modality interventions (Messier et al. 2021; Smith et al. 2023). Furthermore, scalable interventions delivered via telehealth or mobile platforms demonstrated strong potential for accessibility and adherence, especially in contexts where in-person delivery was impractical or resource-limited (Holm et al. 2023; Lawford et al. 2023).

Several studies have also reported barriers to the successful delivery and uptake of interventions. These included limited digital literacy, low motivation or adherence among participants, cultural misalignment of interventions and logistical challenges such as transportation or internet access (Alfieri et al. 2020; Gudbergsen et al. 2021). Conversely, facilitators that enhanced intervention effectiveness included personalised care plans, culturally tailored content, the use of behavioural change techniques and the presence of social or peer support mechanisms (O’Brien et al. 2018; Ojoawo et al. 2024). Studies conducted in low-income and middle-income countries, such as Nigeria and Pakistan, emphasised the importance of culturally relevant and cost-effective programmes to improve feasibility and engagement (Ojoawo et al. 2024; Saleem et al. 2022).

In general, the findings of our scoping review suggest that lifestyle interventions are not only feasible and acceptable but also effective in addressing the multifactorial needs of individuals with knee OA. The results underscore the potential of combining physical, nutritional, psychological and educational components into integrated care pathways to enhance clinical and functional outcomes.

### Quality appraisal

To evaluate methodological rigour, we applied established critical appraisal tools tailored to each study design as demonstrated in Table 2. Of the 36 included studies, 29 were RCTs, two were quasi-experimental, three were observational cohort studies, one was a cross-sectional study and one was a qualitative study.

**TABLE 2:** Quality appraisal of the included studies.

Author (years)	Study design	Appraisal tool	Quality rating	Key appraisal comments
Alghadir et al. (2019)	Randomised controlled trial	RoB 2	Low-to-moderate risk	Randomisation adequate; blinding limited
Alfieri et al. (2020)	Randomised controlled trial	RoB 2	Low-to-moderate risk	Randomisation adequate; blinding limited
Bendrik et al. (2021)	Unclear	Not appraised	Not appraised	Study design unclear or insufficient detail
Bennell et al. (2017a)	Randomised controlled trial	RoB 2	Low-to-moderate risk	Randomisation adequate; blinding limited
Bennell et al. (2022)	Randomised controlled trial	RoB 2	Low-to-moderate risk	Randomisation adequate; blinding limited
Chen et al. (2021)	Randomised controlled trial	RoB 2	Low-to-moderate risk	Randomisation adequate; blinding limited
De Rooij et al. (2017)	Randomised controlled trial	RoB 2	Low-to-moderate risk	Randomisation adequate; blinding limited
Focht et al. (2022)	Randomised controlled trial	RoB 2	Low-to-moderate risk	Randomisation adequate; blinding limited
Gohir et al. (2021)	Randomised controlled trial	RoB 2	Low-to-moderate risk	Randomisation adequate; blinding limited
Gudbergsen et al. (2021)	Randomised controlled trial	RoB 2	Low-to-moderate risk	Randomisation adequate; blinding limited
Godziuk et al. (2023)	Randomised controlled trial	RoB 2	Low-to-moderate risk	Randomisation adequate; blinding limited
Harris et al. (2023)	Unclear	Not appraised	Not appraised	Study design unclear or insufficient detail
Henriksen et al. (2023)	Randomised controlled trial	RoB 2	Low-to-moderate risk	Randomisation adequate; blinding limited
Hinman et al. (2020)	Randomised controlled trial	RoB 2	Low-to-moderate risk	Randomisation adequate; blinding limited
Holm et al. (2023)	Randomised controlled trial	RoB 2	Low-to-moderate risk	Randomisation adequate; blinding limited
Jorge et al. (2015)	Randomised controlled trial	RoB 2	Low-to-moderate risk	Randomisation adequate; blinding limited
Khachian et al. (2020)	Randomised controlled trial	RoB 2	Low-to-moderate risk	Randomisation adequate; blinding limited
Kaufman et al. (2022)	Randomised controlled trial	RoB 2	Low-to-moderate risk	Randomisation adequate; blinding limited
Lange et al. (2009)	Cross-sectional	Newcastle–Ottawa Scale	Moderate quality	Selection adequate; confounders not controlled
Lawford et al. (2023)	Randomised controlled trial	RoB 2	Low-to-moderate risk	Randomisation adequate; blinding limited
Li et al. (2020)	Randomised controlled trial	RoB 2	Low-to-moderate risk	Randomisation adequate; blinding limited
Loeser et al. (2017)	Randomised controlled trial	RoB 2	Low-to-moderate risk	Randomisation adequate; blinding limited
Messier et al. (2021)	Randomised controlled trial	RoB 2	Low-to-moderate risk	Randomisation adequate; blinding limited
Mohammed and Rasool (2023)	Randomised controlled trial	RoB 2	Low-to-moderate risk	Randomisation adequate; blinding limited
Ojoawo et al. (2024)	Randomised controlled trial	RoB 2	Low-to-moderate risk	Randomisation adequate; blinding limited
Rafiq et al. (2021)	Randomised controlled trial	RoB 2	Low-to-moderate risk	Randomisation adequate; blinding limited
Sadeghi et al. (2022)	Randomised controlled trial	RoB 2	Low-to-moderate risk	Randomisation adequate; blinding limited
Sadeghi et al. (2023)	Randomised controlled trial	RoB 2	Low-to-moderate risk	Randomisation adequate; blinding limited
Saleem et al. (2022)	Randomised controlled trial	RoB 2	Low-to-moderate risk	Randomisation adequate; blinding limited
Saw et al. (2016)	Randomised controlled trial	RoB 2	Low-to-moderate risk	Randomisation adequate; blinding limited
Sengul et al. (2022)	Randomised controlled trial	RoB 2	Low-to-moderate risk	Randomisation adequate; blinding limited
Smith et al. (2023)	Randomised controlled trial	RoB 2	Low-to-moderate risk	Randomisation adequate; blinding limited
Somers et al. (2012)	Unclear	Not appraised	Not appraised	Study design unclear or insufficient detail
Strath et al. (2020)	Unclear	Not appraised	Not appraised	Study design unclear or insufficient detail
Walrabenstein et al. (2022)	Unclear	Not appraised	Not appraised	Study design unclear or insufficient detail
Wang et al. (2021)	Randomised controlled trial	RoB 2	Low-to-moderate risk	Randomisation adequate; blinding limited

Note: Please see the full reference list of the article, Bello, B., Aliyu, F.Y., Kaka, B. & Useh, U., 2026, ‘Exploration of lifestyle interventions for individuals with knee osteoarthritis: A scoping review’, *South African Journal of Physiotherapy* 82(1), a2370. https://doi.org/10.4102/sajp.v82i1.2370 for more information.

RoB 2, Cochrane Risk of Bias 2.0.

For the RCTs, the Cochrane Risk of Bias 2.0 tool was employed (Sterne et al. 2016). Most RCTs demonstrated low to moderate risk of bias with clear randomisation procedures and pre-specified outcomes (Bennell et al. 2017a; Focht et al. 2022; Smith et al. 2023). However, several trials, particularly those involving exercise programmes, lacked blinding of participants and personnel, a common limitation in behavioural interventions. Moreover, some studies reported high attrition, with over 20% dropout, introducing the risk of bias in outcome reporting (Hinman et al. 2020). Quasi-experimental studies were evaluated using the Joanna Briggs Institute (JBI) Critical Appraisal Checklist for non-randomised designs (Moola et al. 2020). These studies often reported strong pre-test and post-test measures and plausible causal pathways (Østerås et al. 2021). Nonetheless, the absence of control groups and unaccounted confounders were common issues that could weaken internal validity.

The Newcastle–Ottawa Scale was applied to cohort and cross-sectional studies (Wells et al. 2000). These studies generally scored well on participant selection and outcome ascertainment, yet many failed to adjust for potential confounding variables or report comparability between groups (Alfieri et al. 2020; Alghadir et al. 2019). The only qualitative study was appraised using the Critical Appraisal Skills Programme (CASP) Qualitative Checklist (CASP 2018). While most studies demonstrated credibility and data transparency, a few lacked explicit discussions of author reflexivity or sampling justifications. Across all study designs, recurring limitations included small sample sizes, short intervention durations, a lack of long-term follow-up and minimal exploration of socio-economic or cultural context. Nevertheless, the frequent use of validated tools such as WOMAC, SF-36, and pain rating scales, as well as theory-driven intervention frameworks, enhanced confidence in the reported findings. Future trials would benefit from stronger reporting of implementation fidelity, greater attention to equity and inclusion of diverse populations.

## Discussion

Our scoping review revealed the growing body of evidence supporting multimodal lifestyle interventions as effective strategies for managing knee OA. In our review, lifestyle interventions are defined as structured or semi-structured, non-pharmacological strategies aimed at modifying individuals’ daily behaviours to improve health outcomes in knee OA. These interventions typically target modifiable risk factors and functional limitations through approaches such as physical activity and exercise, weight management, dietary modification, patient education, and behavioural or psychological strategies, including self-management training and cognitive behavioural approaches (Bannuru et al. 2019; Hunter & Bierma-Zeinstra 2019). This definition reflects contemporary models of care that emphasise patient-centred and sustainable approaches to managing knee OA.

Our review synthesised findings from 36 studies, involving 4025 participants, spanning diverse settings and populations, revealing that programmes incorporating multiple lifestyle components, such as exercise, weight management, education, psychological support and behaviour change techniques, were more effective in improving pain, physical function and quality of life compared to single-component interventions (Bennell et al. 2020; Hinman et al. 2020; Messier et al. 2022). The results demonstrate that lifestyle management for knee OA has evolved beyond simple exercise prescriptions. While traditional physiotherapy-guided exercise remains a foundational element, there is a significant trend towards incorporating psychological and educational components. Interventions such as cognitive behavioural therapy, mindfulness-based Tai Chi and pain coping skills training were prominent in the literature. This shift aligns with the biopsychosocial model of chronic pain, suggesting that addressing the psychological burden of OA is as critical as addressing physical dysfunction. The synergistic effect of combining structured exercise with dietary advice and self-management education was repeatedly demonstrated across the studies (Chen et al. 2021; Lawford et al. 2018). Interventions that adopted behavioural coaching models or digitally supported platforms were especially effective in enhancing patient engagement and adherence, a critical determinant of long-term outcomes (Smith et al. 2023; Walrabenstein et al. 2022). These findings align with the biopsychosocial framework of chronic disease management, emphasising that sustained improvements in OA symptoms require addressing biological, behavioural and contextual factors (Loeser et al. 2017). A notable finding is the proliferation of digital health interventions, including mobile apps, tele-physiotherapy and web-based platforms. These models appear to offer clinical benefits comparable to in-person care while providing greater flexibility. However, the identification of ‘digital literacy’ and ‘tech usability’ as recurring barriers suggests that while eHealth increases accessibility for some, it may create new barriers for older populations or those with limited technological proficiency. Considering the diversity of intervention models and populations studied, future research should adopt more standardised, yet simple and pragmatic, outcome measures.

Our review highlights tools such as the TUG test, 30-s sit-to-stand test and patient-reported global rating of change as feasible alternatives to comprehensive instruments like WOMAC and KOOS, particularly for large-scale community or digital trials (Alghadir et al. 2019; Kaufman et al. 2022). Additionally, measures of patient activation and self-efficacy (e.g. Patient Activation Measure [PAM]-13) provide valuable insights into behaviour change, which is crucial for lifestyle modification (Lawford et al. 2023). Adherence to lifestyle interventions emerged as a key determinant of effectiveness across the included studies, with several interrelated factors influencing compliance and participant engagement. Motivation was frequently reported as a central barrier, particularly in programmes requiring sustained behavioural change such as exercise adherence, dietary modification or self-management practices (Giardulli et al. 2025). Additional challenges included limited access to facilities or technology, time constraints, low digital literacy in eHealth-based interventions and variability in individual readiness for change (Ojoawo et al. 2024). Conversely, facilitators of adherence included structured, supervised programmes; personalised or tailored interventions; ongoing coaching or professional support; and the incorporation of social or peer support mechanisms. Interventions described as ‘holistic’ in our study typically referred to multicomponent programmes that combined physical (e.g. exercise therapy), behavioural (e.g. education and self-management strategies) and, in some cases, psychosocial or dietary elements. These integrated approaches appeared to enhance engagement by addressing the multifactorial nature of knee OA and supporting sustained behaviour change. Collectively, these findings highlight that beyond intervention content, the design and delivery context play a critical role in influencing adherence and overall outcomes.

A primary objective of our review was to identify the outcome measures used to evaluate lifestyle interventions in individuals with knee OA. The findings demonstrate considerable heterogeneity, with no single outcome measure consistently applied across all included studies. Although the WOMAC remains the most frequently utilised tool for assessing pain, stiffness and physical function, its continued prominence in recent trials highlights its central role in evaluating clinical outcomes (Ali et al. 2025). Similarly, contemporary interventions continue to incorporate broader outcome domains, including health-related quality of life using the SF-36, which has shown significant responsiveness to lifestyle interventions (Chen et al. 2021), and performance-based measures reflecting functional mobility.

This diversity reflects the multidimensional nature of knee OA and the broad targets of lifestyle interventions, which encompass physical, psychological and behavioural components. Recent evidence confirms that exercise, weight management and combined lifestyle programmes improve pain, physical function and overall well-being, although the magnitude of clinical benefit may vary across settings (Huffman et al. 2024; Landry & Bagha 2026). Moreover, emerging models of care continue to integrate physical activity and behavioural strategies as first-line interventions, reinforcing the need to capture multiple outcome domains (Izquierdo, Ramírez-Vélez & Fiatarone Singh 2025). Despite these advances, methodological challenges persist. The variability in outcome selection limits comparability across studies, complicates evidence synthesis and restricts the feasibility of robust meta-analyses. Wu et al. (2022) highlight the ongoing difficulties in evaluating the effectiveness of interventions due to inconsistent measurement approaches and undefined key outcome dimensions in knee OA research. The findings of this systematic review highlighted the need to develop and adopt a Core Outcome Set (COS) tailored to lifestyle interventions for knee OA. Standardisation of outcome measures would enhance comparability, strengthen evidence synthesis and facilitate translation into clinical practice. Importantly, future COS development should incorporate stakeholder perspectives, including patients and clinicians, to ensure that selected outcomes are meaningful, comprehensive and aligned with real-world care priorities.

### Strengths and limitations

A major strength of our review lies in its comprehensive scope and methodological rigour, which involved systematic identification, screening and data extraction across diverse databases and study types. The inclusion of various research designs, including RCTs, qualitative studies and quasi-experimental designs, provided a holistic understanding of the intervention landscape. Furthermore, our review captured global perspectives, with studies from both high-income and resource-limited settings, providing a broad understanding of contextual factors. However, several limitations should be acknowledged. Firstly, despite efforts to include diverse sources, most studies were conducted in high-income countries, which may limit generalisability to low-resource settings. Secondly, heterogeneity in intervention components, delivery formats and outcome measures across studies impeded direct comparison and meta-synthesis. Thirdly, many studies reported short-term outcomes, leaving questions about the sustainability of observed benefits. Finally, publication bias cannot be ruled out, as grey literature and non-English studies were excluded, which may have omitted relevant interventions implemented in non-academic contexts.

## Conclusion

Lifestyle interventions for knee OA are diverse and generally effective in improving pain, function and quality of life. However, substantial variability in intervention design and outcome measures limits comparability and evidence synthesis. Standardising outcome assessment through a COS and improving consistency in reporting will strengthen future research and support the translation of lifestyle interventions into clinical practice.
